# A comprehensive review on cardiovascular disorders development due to salt intake: an emphasis on policy implementation

**DOI:** 10.1186/s12961-025-01305-z

**Published:** 2025-03-11

**Authors:** Ian Osoro, M. G. Rajanandh

**Affiliations:** Department of Pharmacy Practice, SRM College of Pharmacy, SRM Institute of Science and Technology, Kattankulathur, Chennai, Tamil Nadu 603203 India

**Keywords:** Salt reduction, Sodium, Policies, Population, Cardiovascular diseases, Hypertension

## Abstract

Excessive salt consumption has been linked with the emergence of hypertension, which further leads to cardiovascular disease development among other medical conditions. This has resulted in leading world institutions such as the WHO coming up with relevant plans to minimize its use. Lower–middle-income countries (LMICs) have greatest burden of noncommunicable diseases (NCDs), with hypertension being a common condition. Reduction of salt intake is a great control measure in minimizing the rise in prevalence of hypertension or cardiovascular diseases. Many countries have agreed and even formulated their salt reduction policies as recommended by the WHO, however, the challenge is widely noted in implementation. Thus, few countries have been able to achieve the global WHO recommended standards of daily salt intake. Salt is the main source of sodium in our diets, which is an essential component responsible for the balance of the extracellular fluid volume but may lead to salt-induced hypertension when used excessively. The achievement of salt reduction is predicated on multiple factors such as knowledge, attitude and practice of the public. Therefore, localizing interventions with strategies such as public media campaigns, reformulation of processed foods (mandatory and voluntary) and front-of-packaging labelling awareness. Some of the reasons for failure in implementation include economic challenges, lack of visionary leadership, stakeholder struggles and poor planning and execution of strategies. This review aims to elaborate on the development of cardiovascular diseases or hypertension due to salt usage and the recent advancement regarding salt reduction policies. Further, we assess the need for proper implementation with the United Kingdom as a case study. In conclusion, most governments have made the right decisions in developing or recommending salt reduction strategies to the food industry. However, more focus is needed to ensure effective implementation of the plans.

## Introduction

Cardiovascular diseases (CVD) are the world’s leading cause of deaths, with an estimate of 17.9 million cases yearly. Lower–middle-income countries are most affected, with about 80% of total CVD deaths occurring in these regions [1]. The trajectory of CVD prevalence is on an upwards curve, with an estimate of 184 million cases globally of CVD with hypertension by the year 2050 [2]. Notably, women experience more fatal outcomes compared with men, and in the United States, about 60 million women have at least one CVD [3]. Further, Black women are at a higher risk (60%) of developing CVDs than white women [4]. Currently, processed foods, bread products, meat, cereals and dairy products are the most common sources of dietary salt. In the United States and other Western countries, it was estimated that 25–40% of salt comes from baked products [5]. Soups, bacon, shrimps, pizza and cottage cheese are some of the ordinary examples of foods high in salt content [6]. High dietary salt intake has been linked with emergence of cardiovascular disorders, particularly hypertension. Various mechanisms such as increased renin angiotensin system, increased endothelial function and inflammation have been postulated to be involved in this pathophysiology [7].

In most domestic settings, salt is added to “taste” with limited consideration of the health outcomes except in specific health conditions such as hypertension, kidney disease and diabetes [8]. The amount of salt to be consumed is overly viewed as a personal choice, whereas the effects are far more devastating. Overweight, alcohol intake, tobacco use, salt-heavy diet and lack of exercise are among the most common behaviours linked to both hypertension and cardiovascular diseases (CVDs). Hypertension affects about 1.5 billion people globally and it has been postulated that blood pressure (BP) is the principal risk factor for CVD [9]. Only in 2019, about 10 million global deaths were attributed to hypertension, whereas 17.9 million deaths were due to CVDs. The WHO recommends a daily salt intake of less than 2000 mg; however, many people consume about 9–12 g daily [10]. A study by Jackson et al. revealed that 86% of their participants diagnosed with hypertension consumed about 2300 mg of salt in the United States [11]. Further, Mente et al. showed that an increase in salt intake was associated with a rise in systolic blood pressure (SBP) among patients with hypertension [12]. Proper management of these health behaviours such as lowering alcohol consumption, physical exercise and weight reduction has been proven to reduce the risks and outcomes [13].

The WHO launched its “Signature Initiative to reduce cardiovascular disease through salt reduction and hypertension control” on 9 December 2022. This is among its six innovation techniques in controlling noncommunicable diseases in Europe [14]. The alarming realities about salt intake are showcased in an article by the WHO on salt reduction, which was published on 29 April 2020 [14]. The report provided information about salt reduction recommendations; a brief overview of salt, potassium and sodium; how to reduce salt in the diet; and misperceptions about salt. It is also important to mention that the WHO has already handled similar cases earlier on, in which they came up with specific guidelines such as “sodium intake in children in 2012” [15].

The launching of this new initiative comes at the right season, especially with the quest for clinical evidence in the prevention and management of CVDs, which have caused havoc globally in the past. European nations with high CVD rates are poised to become the first beneficiaries of this initiative. With the target of reducing salt intake globally by 30% by 2025, this will be the right pathway going forward. Rational changes in healthcare policy have been proven to bring positive outcomes. For example, the introduction of the India Hypertension Control Initiative (IHCI), which was launched in 2017, has shown improvement in the control rate of hypertension [16].

Some of the few LMICs that have adopted salt reduction policies include South Africa, Argentina, Mongolia and Vietnam. Globally, most countries agree with salt reduction, however, the implementation of these policies has not been a smooth path. A fundamental misunderstanding among individuals is whether salt intake should be based on individual decisions or whether the government should strictly enforce lowered salt intake in the diet. However, this misunderstanding is caused by misconceptions about salt intake. For example, many believe that salt intake comes from the food prepared in their homes, whereas in reality, above 70% of the salt intake comes from processed foods [17]. Another misunderstanding is that there will be a salty taste in foods high in salt. This is completely wrong since masking the taste using flavouring agents may occur, hence checking for the sodium levels is the best way [18].

Some setbacks have been seen, for example, in the United Kingdom where the change in government led to the derailment of the salt reduction policy [19]. Such loopholes, in addition to others, can therefore be noted when there is a change in government, but a resolution to maintain the correct standard of health despite political changes should be able to overcome this instability. The enrolment of a firm independent food agency free from any political bias will therefore prove invaluable. Further, challenges such as limited resources significantly impede the successful implementation salt reduction strategies. Thus, we decided to conduct this review to discuss effective deployment salt reduction strategies with a focus on cardiovascular disease development and policy implementation.

## WHO efforts towards salt intake reduction

Regional disparities on salt consumption have been noted in areas such as East Asia when compared with Northern America whereby in the former region, salt consumption is relatively higher than in the latter [20]. This ultimately corresponds with the linear correlation between salt consumption and CVD mortality rates in both regions, where those with high salt intake have more deaths and vice versa [21]. The idea that salt amounts in the foods we consume should not be regulated is challenged in the “Salt intake report”. The report sets out why sodium and potassium levels which are important in our body can turn out to be harmful and why salt reduction will improve the health status of individuals. The overall positive outcomes of salt reduction include 2.5 million lives being saved annually, the risk of heart disease being highly reduced and a higher chance of longer life when we reduce the salt intake as desired across all borders.

Strategies that contribute to the reduction of salt in food include creating government policies that advocate for affordable and healthy products, raising awareness among consumers on the benefits of salt reduction, monitoring the salt intake of the population and accessing the knowledge of the consumers about salt consumption and inculcating salt reduction programs within the systems such as schools, so on. The high number of CVD deaths is a reminder of the urgency needed in salt reduction [22]. The salt intake reduction strategy is presented as a new initiative that will help reduce salt consumption at home. This signature initiative is not focussing on salt in processed foods but rather aims at reducing salt intake on an individual level, which ultimately improves hypertension management. The specific intention of this initiative is simple: “your salt consumption affects your health more than you might have previously known”. This initiative comes as a complement to the salt reduction package produced by WHO/Europe in July 2021. It is a part of the WHO Action Network on Salt Reduction in the Population in the European Region, which involves around 20 countries. Another additional five strategies are expected to be rolled out among these countries in response to achieving salt reduction among the member states.

**Fig. 1:**
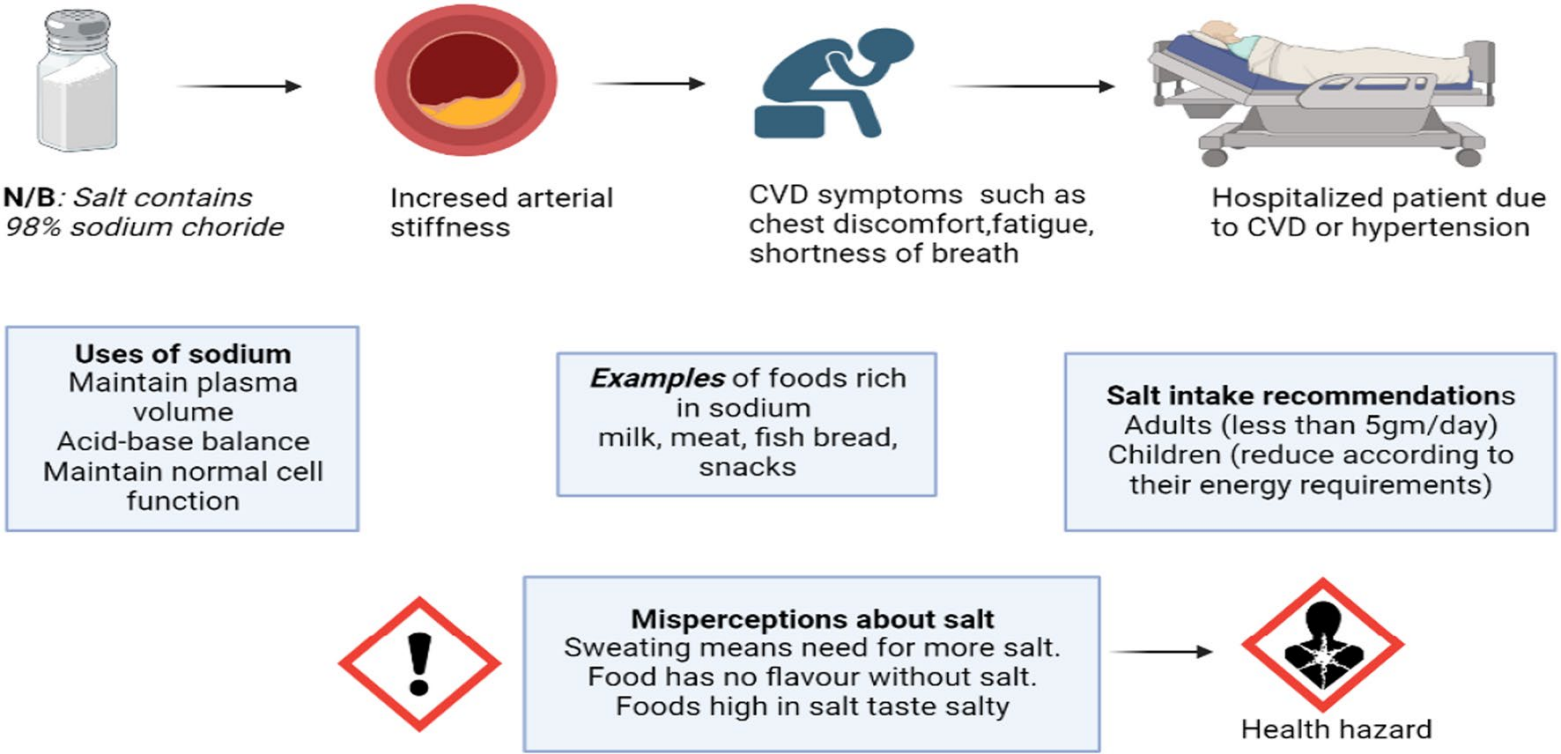
Effect of excessive salt on the human body

The long-term vision of these initiatives is to reduce salt consumption by 30% by 2025, paving the way for a possible revolution worldwide in salt consumption. The realization of these initiatives will require proper support and willingness to adapt to this new lifestyle from the community. Strict implementation of the initiatives will provide a platform to understand the real effect of modifications on individual salt consumption. The planned tracking of the WHO Salt Reduction 2025 plan will further improve the quality of life and increase the lifespan among patients with hypertension and CVD. High salt intake has been associated with medical conditions such as high blood pressure, cardiovascular disease, osteoporosis, stomach cancer, kidney disease, renal stones and obesity [23]. Figure 1 below illustrates succinctly the importance of sodium, the consequences of excessive salt, and the misperceptions about salt.

## Salt intake and cardiovascular disease

Many studies conducted have shown that a high intake of salt leads to CVD development. It has been established that high salt intake usually results in hypertension, which ultimately leads to cardiovascular disease development [24]. Salt is a combination of 40% sodium and 60% chloride. This high amount of sodium chloride is linked with elevated blood pressure in the human body as compared with when other compounds such as sodium bicarbonate are used. Salt is the main source of sodium in our diets, which is an essential component responsible for the balance of the extracellular fluid volume ultimately affecting the blood pressure level in the human body [25].

In a meta-analysis by Rios-Leyvraz et al., children with lower salt intake were found to have lower systolic BP than their counterparts after follow-up [26]. This further proves that the reduction of salt intake at early life stages plays a crucial role in the expected outcome during adulthood. Minimal intake of sodium in children was seen to have a positive outcome [27–29]. The majority of countries with high sodium intake have been seen to have a higher prevalence of hypertension [30, 31]. In a meta-analysis study by Strazullo et al., it was discovered that salt intake had a positive correlation with the emergence of stroke and other cardiovascular complications. This study included 177,025 participants from 13 studies and the authors recommended salt reduction as a measure of reducing the rising incidence of stroke and CVD complications [32]. It has been noted that the positive correlation between salt intake and hypertension usually occurs over a significant period, hence younger individuals tend to have their blood pressures less affected by high salt intake as compared with older age individuals, and part of this can be attributed to the weakened body system [33].

This necessitates the need to develop and implement good policies on salt reduction from which the older populations will benefit most [34, 35]. There have also been a few contradicting studies showing little evidence of this occurrence, however, it was noted that most of these studies were short term. Moreover, these studies had used intensive methods (i.e. reducing the salt consumption levels to very low) in curbing salt consumption, thereby the results produced could have been biased [36–38]. Therefore, it is widely accepted among healthcare practitioners that salt consumption has to be minimized, particularly in individuals whose health is at risk. Besides hypertension, high dietary salt intake has been discovered to play a role in several other diseases, such as stroke, kidney failure, and so on. [10, 39].

On the basis of data from stroke mortalities in the United Kingdom, it has been revealed that reducing the salt intake to 6 g/d is likely poised to prevent about 2.5 million deaths yearly [40, 41]. However, it should also be noted that the impact of salt in Chronic kidney disease (CKD) and stroke is independent of a patients hypertension status. This is because of the varying underlying mechanisms of CKD and stroke [39]. Embedding lifestyle changes with salt reduction has been seen to reduce the cases of heart attack, hence it is advisable to inculcate both of these habits [42]. Additionally, salt reduction to levels below 2.14 g/day has been associated with an increase in cardiovascular risk, hence even with minimization, care should be taken [43]. Other studies have shown that individuals with a high risk of CVDs and having sodium excretion are more likely to undergo severe myocardial infarction and their mortality rates are higher [44, 45].

## Salt-induced hypertension

Salt-induced hypertension/salt-sensitive hypertension is a physiological characteristic exhibited by animals where the salt intake directly influences the blood pressure levels proportionally in an organism, which is a result of salt sensitivity. Both animal and human studies have shown this to be true [46]. Despite high salt intake being associated with rise in BP, it has been noted that some individuals have a greater salt sensitivity than others. Such individuals are termed to be salt sensitive since they display a higher BP when they have taken excess salt and vice versa. On the contrary, those individuals in whom there is no greater disparity due to salt intake are referred to as salt resistant [47].

Epidemiological figures show that salt sensitivity occurs in 25% of individuals with normal BP and nearly 50% of patients with hypertension [48]. There have been two known mechanisms for salt sensitivity development based on time frames, that is, acute and progressive. The acute development mechanism occurs when the surge in BP comes almost immediately after high dietary salt intake, whereas for the progressive mechanism, it takes slower and rather longer periods before the effect on BP is seen. Moreover, acute development is usually reversible, whereas progressive development is mainly irreversible [49]. Sex, presence of comorbidities (i.e. hypertension, kidney conditions), and genetic make-up are some of the predisposing risk factors associated with salt sensitivity development. Moreover, obesity and female sex have been linked with higher salt sensitivity [50].

Salt sensitivity currently has no diagnostic criteria, thereby making it hard to detect. However, two protocols (inpatient and outpatient) have been developed to enable clinicians to diagnose it. For the outpatient protocol, the patient is expected to strictly use low and high salt dietary intake in the first week and second week, respectively. On the basis of the outcome, the individual is categorized as either salt sensitive or salt resistant. In the inpatient protocol, it is required to note when there is an increase in the extracellular volume [normal saline intravenous (IV) administration] on day one. Later, furosemide accompanied by a low salt dietary intake is used to minimize the volume. Although both methods are usable, the outpatient system has more preference because it can ultimately be used in CVD prediction [51, 52].

## Development mechanisms of salt-dependent hypertension

Table 1 gives a summary of the pharmacological development of salt-dependent hypertension (HTN).

**Table 1:** Summary of pharmacological development of salt-dependent HTN

Mechanism	Pathophysiology	References
RAAS activation	An overactive RAAS system leads to vasoconstrictor and antidiuretic effects on the body. This ultimately leads to increased systemic resistance and thereby elevated BP levels	[53, 54]
Aldosterone related signaling	Aldosterone activates mineralocorticoid, which enhances sodium reabsorption. This progression leads to the development of BP	[55, 56]
Sympathetic nervous system (SNS) disruption	Renal denervation is poised to cause elevated BP. This occurs whenever the SNS is activated, causing renal antinatriuresis through stimulating renal secretion, reduced kidney perfusion and increased tubular sodium reabsorption	[57, 58]
Vasodysfunction theory	Failure to retain maximum amounts of sodium in the body leads to vasodilation, thereby causing an increase in the blood pressure	[59, 60]
Immune system activation	Increased RAAS and SNS cause the MDSC and Treg cells to try to suppress inflammation. Inflammasomes, together with the gut microbiome, acts on the dendritic cells, leading to HTN progression	[61–63]

## Interventions thus far from global to local/governmental

The achievement of salt reduction is predicated on multiple factors such as knowledge, attitude and practice of the public. Therefore, localizing interventions amongst populations has been seen to be a better way of addressing salt reduction, along with enhancing government regulatory policies. Ghimire et al. revealed that in South Asia, proposed salt reduction policies are yet to be implemented properly and proposed that more community health promotion studies should be conducted to enhance reduction of salt usage [64]. On the other hand, Jordan et al. revealed that having a tailored salt reduction program in communities would enhance the effectiveness of the program [65]. In concurrence, Land et al. conducted an integrated community-based program in a town in Australia where a 10% reduction was noted on the basis of the intervention [66].

Further, LMICs have been most affected by salt-induced hypertension/CVD, hence the need to ascertain best practices that can be tailored for these nations [67]. A study by Webster et al. which assessed four LMICs (Mongolia, South Africa, Vietnam and Argentina) showed that having proper knowledge of the sources of salt and requisite funds to implement policies, along with collaboration between various stakeholders and effective leadership, could be very crucial in lowering salt intake [68]. Another study conducted in the sub-Saharan Africa (SSA) region showed that the progress of salt reduction is quite slow within the region, with lack of resources and other health priorities being the main barriers [69].

Moreover, LMICs currently have the highest rate of fatalities from noncommunicable diseases (80%) [70]. Poor management and treatment of hypertension has particularly played a key role in this occurrence [71]. Additionally, dietary patterns have shifted from traditional to processed foods, for instance, in the Democratic Republic of Congo, where bread has replaced cassava [72]. In Nigeria, bread is taken as a meal or snack instead of other traditional meals [73]. Some of the factors responsible for this shift are changing food preferences, affordability, easy accessibility, time conservation and urbanization/westernization mainly spread through social media [74]. Therefore, understanding the peculiar nutrition context of many LMICs will act as a key springboard in determining which steps should be implemented to reduce salt consumption [75].

Apart from the WHO, many global cardiovascular societies have campaigned for the reduction of salt strategies within their various jurisdictions, such as the American Heart Association, European Cardiovascular Society, Heart and Stroke Foundation of Canada and Cardiology Society of India [76–79]. Lowering salt consumption has been noted to have several beneficial effects on human health, such as lowering risk of cancer, hypertension and other CVD development. Further, high risk of chronic kidney disease has been associated with high salt intake, hence it is important to minimize its consumption particularly amongst these patients [80]. In addition, lowering salt intake levels has been seen to reduce the risk of gastric cancer [81]

## Studies on the implementation of salt reduction policies with relation to CVD—UK case study

The policies adopted by any nation happen to be the roadmap that shapes future outcomes. Conducting community-based salt reduction programs has been complex, however, the resulting outcomes have been promising. To develop an effective intervention/strategy, a lot of cooperation is needed between various key players such as the consumers, healthcare practitioners, producers and the government. A case study is the United Kingdom, which happens to be one of the leading countries to have had a successful campaign, and other countries are emulating their steps [82, 83]. The Scientific Advisory Committee on Nutrition for the United Kingdom (SACN) assessed the relationship between how salt is linked with CVDs, and they concluded that reducing salt consumption from 9 g/d (as seen in a 1994 study) to 6 g/d would significantly reduce hypertension and cardiovascular diseases. On this basis, both the Health Department and Food Standards Agency (FDA) set up the goal of reducing the salt consumption of each household to 6 g/day. In the 2000–2001 period, the average salt intake was 9.5 g/day, however, after implementing reduction strategies, the average had reduced to 8.6 g/day in 2008 [84]. The strategies used by the United Kingdom are public media campaigns, reformulation of processed foods (mandatory and voluntary) and front-of-packaging labelling awareness.

## Public media campaign

In September 2004, the first salt reduction campaign was launched, with the main message focussing on the negative effects of high salt intake. Subsequently, another campaign was conducted approximately a year later with the main message focussing on the need for consumers to read the salt levels on food labels. In March 2007, a third episode was carried out to inform the public of the need to watch the foods they consume since the majority already contained enough salt. Materials such as leaflets were also used in these campaigns to cover a wider population [85].

Furthermore, Public Health England (PHE) initiated the Change4Life (C4L) program in 2009, which was aimed at creating greater public awareness of the need to avoid consuming foods rich in salt and sugars and generally reduce obesity. This campaign ran through different platforms, that is, television, print media, etc., and it was meant not only to inform the public, but also to convince them of the need to observe proper diet. Building a consistent eating pattern, eating more vegetables and minimizing taking foods rich in sugar were some of the healthy eating patterns. Additionally, observing body weight and regularly engaging in physical exercise was encouraged in the advertisements.

**Fig. 2:**
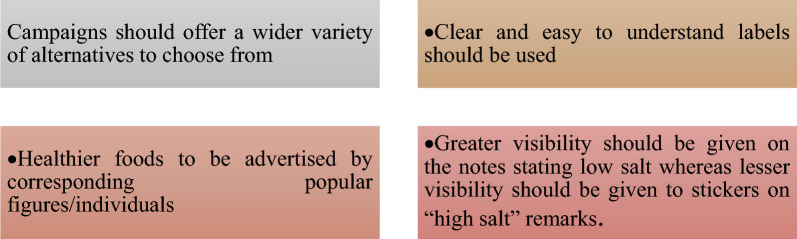
Recommendations for making the use of public media more effective in health-related campaigns

Results from these campaigns have been positive since it was noted that more consumers were aware of the 6 g/d and there was an increase in the number of individuals trying to intentionally minimize their salt intake [86, 87]. The use of a public media campaign as a strategy comes along with challenges such as the unidirectional nature of the communication. However, with proper planning and understanding of the target group, these challenges can be overcome. The use of media in health campaigns has proven to be effective, hence the need to focus even more on this strategy using strategies shown in Fig. 2 [88].

## Reformulation of processed foods

The process of redeveloping manufactured food products to make them both safer and healthier is referred to as food reformulation. This practice is mainly intended to reduce the salt, sugar or fat content in a product. Ultimately, the proper implementation of food reformulation practices and policies is aimed at reducing the development of noncommunicable diseases in the public [89]. In a study by Gressier et al., it was discovered that the reformulation of food products that happened in the United Kingdom (in the salt reduction strategy) played a vital role in the overall reduction of salt consumption among individuals between 2008 and 2017 [90].

A meta-analysis study by Jaenke et al. showed that a 40% reduction of salt in bread and 70% in meat will not significantly affect the consumer acceptance of the product. However, disparities were noted among the various reformulated products, hence the need for creating proper awareness that will enhance understanding [91]. Some of the challenges in the reformulation of processed foods include the need for more ingredients, the use of ingredients unfamiliar to the consumers, the need for more warnings on packages, the possibility of compromise of the product and using different ingredients that may affect the product’s quality and shelf life.

## Front-of-packaging labelling (FOPL) awareness

Food labelling has been an essential part of processed foods, however, its effectiveness thereof has not been great since much of the information is written on the package, which many times requires calculations. This thereby prevents consumers from making a well-informed decision with regard to quantities of nutrients, for example, salt and others. [92–95]. The FOPL system is aimed at relaying information simply and effectively regarding the excessive vital nutrients in a product, such as salt, fats, sugars and so on. The use of nutritional labels has been linked to consumption of healthy foods, however, using poorly designed labels is likely to be ineffective [96–101]. Most of the FOPL-related studies were associated with sodium or sugar [102–108]. Effective FOPL enables the consumers to anchor on relevant health-associated information. Therefore, countries should develop good nutrition labels that are easy to understand and relatable to the community.

Some countries have been able to implement the use of nutritious labels in a voluntary or mandatory manner. In voluntary contexts, it was noted that the firms chose to have their products labelled healthier to attract more consumers. Additionally, it was discovered that implementing a mandatory system would be more effective as compared with having a voluntary system of labelling products. Worthy of note is that those products having labels were generally scored as healthier compared with the unlabelled ones [109–112].

## Importance of having well-established salt reduction policies

The period between 2003 and 2014 happened to be the peak of the salt reduction strategy in the United Kingdom after the efforts had resulted in a 19% salt reduction (from 9.38 g/day to 7.58 g/day) in the population. This translated to more than 9000 lives saved [113]. Unfortunately, the government handed over the responsibility of salt reduction to the manufacturers, where the implementation has largely been a failure. Transferring authority from the FSA to the Department of Health in 2010 brought confusion about who is in charge, thereby forcing the minister of health to develop a responsibility deal where the food industry would be in charge of policy implementation.

**Fig. 3:**
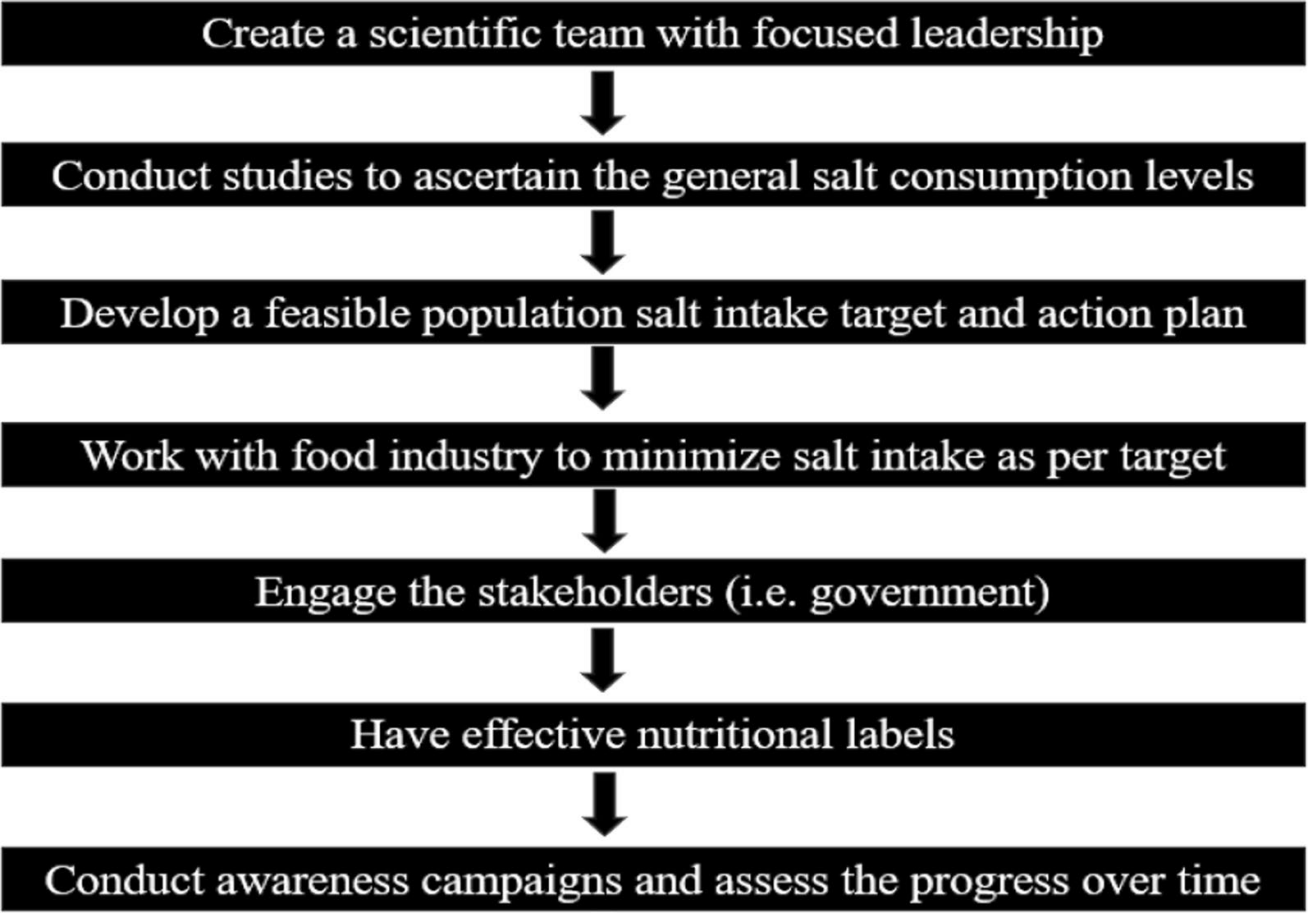
How to create a salt reduction strategy

This caused many nongovernmental organizations to quit, stating that the new direction being taken was meant to prioritize food industries over general public health [114]. Even with the introduction of a new minister and proposed changes, the salt reduction strategy was unable to meet its aims with challenges from funding to lobbying and even unequal competition. The rising incidence of hypertension and other health-related challenges is a testament to the failing system [114]. In a study by Jing et al., a rise was seen in salt consumption to 8.39 g/day in 2018. Additionally, it was estimated that 38,000 stroke and cardiac conditions could have been prevented. A 1.45 g/day reduction would have been noticed between 2014 and 2018 had the program proceeded as intended [115]. However, in a recent news article, a government spokesperson said the salt reduction program has been successful in reducing salt intake by 20% as of 2006 [116]. Therefore, to enhance the robustness and efficacy of salt reduction policies, Fig. 3 below gives a proper way of developing salt reduction strategies.

## Developing a salt reduction strategy

### Limitations

It is important to note that our study has some limitations. Firstly, long-term health outcomes of salt reduction policies remain underexplored due to limited longitudinal studies found. Secondly, only literatures in English language were assessed, which may thereby omit other relevant studies published in other languages. Further, there could have been potential biases in the selected studies since it is a narrative review. In addition, there is reliance on secondary data without primary investigation.

## Conclusions

Salt reduction strategies conducted over a prolonged duration have shown effectiveness in enhancing the population’s health. This should thereby encourage more responsible authorities to take up relevant actions geared towards minimizing salt consumed by humans. Adopting practices such as conducting regular reviews on the compliance of the food industry regarding salt reduction will prove to be crucial. The efforts may seem not to significantly reward immediately, however, after some time, the public will enjoy the outcomes from this preventive strategy.

## Future perspectives

Despite the challenges, the salt reduction program remains one of the most efficient health campaigns worldwide. Embracing new solutions and resolutions can cause the strategy to become even more effective as there is already a pathway to follow. Having an independent body overseeing the salt reduction would be the best way to ensure that all stakeholders move towards a similar direction in unity. Mandatory implementation of salt reduction policies should be adopted rather than a voluntary system to enable equal market competition [117, 118].

## What does this paper contribute to the wider global clinical community?

- It shows the need for having proper implementation of healthcare policy guidelines
- It gives a clear view of the importance of enhancing good governing systems for healthcare

## Data Availability

No datasets were generated or analysed during the current study.

## Abbreviations

RAAS: Renin angiotensin aldosterone system
SNS: Sympathetic nervous system
BP: Blood pressure
HTN: Hypertension
CVD: Cardiovascular diseases
WHO: World Health Organization
FOPL: Front-of-packaging labelling
MDSC: Myeloid-derived suppressor cells
